# Applying nephelometry for analyzing liquid yeast cultures

**DOI:** 10.1016/j.bbrep.2026.102572

**Published:** 2026-04-02

**Authors:** Laura Tünnermann, Justine Colou, Torgny Näsholm, Tommy Löfstedt, Regina Gratz

**Affiliations:** a Department of Forest Genetics and Plant Physiology, Umeå Plant Science Centre, Swedish, University of Agricultural Sciences, Umeå, 90183, Sweden; b Department of Forest Ecology and Management, Swedish University of Agricultural Sciences, Umeå, 90183, Sweden; c Department of Computing Science, Umeå University, Umeå, 90187, Sweden

**Keywords:** Nephelometry, Spectrophotometry, *Saccharomyces cerevisiae*, Lag time, Maximum slope time, Lysine histidine transporter 1 (LHT1)

## Abstract

*Saccharomyces cerevisiae* is a widely used model organism for the molecular analysis of genes and proteins. Several methods have been developed to study protein function and activity through heterologous gene expression, including yeast two-hybrid and yeast complementation. Traditionally, these yeast-based assays were performed on solid agar plates. While this approach provides an easy visual readout, it is difficult to quantify the results accurately. To overcome this limitation, liquid-based methods were introduced. Most of these methods rely on the use of spectrophotometry to measure reduction in light transmission as a result of light scattering and monitor culture growth.

In this study, we propose nephelometry as an additional method for performing and analyzing liquid-culture yeast complementation assays. More specifically, we compare the suitability of using nephelometry for the functional analysis of two homologous proteins using yeast complementation: The amino acid transporter homologues *Arabidopsis thaliana* LYSINE HISTIDINE TRANSPORTER 1 (AtLHT1) and *Populus tremula L. x tremuloides Michx* LYSINE HISTIDINE TRANSPORTER 1.2 (PtrLHT1.2). In previous reports, no differences in microbial growth were detected, irrespective of which homolog was used to rescue an amino acid-deficient yeast mutant strain. By using nephelometry to record yeast growth, we demonstrated that it is a robust and reproducible method. When comparing to spectrophotometric measurements of yeast cultures, it proved to be a suitable alternative.

The novel approach even revealed previously undetected differences in culture growth of both homologues, highlighting nephelometry's potential to improve sensitivity in yeast-based functional assays.

We present the use of nephelometry as an equal method to yeast complementation traditionally executed on solid agar medium or in liquid culture with spectrophotometric analysis.

## Highlights

• Nephelometry presents an alternative to classic spectrophotometry.
• Nephelometry can be used to characterize growth of liquid yeast cultures.
• Semi-automatic production of results which are stable and reproducible.
• Comparing AtLHT1 and PtrLHT1.2 shows significant differences in maximum slope time.

## 1 Introduction

*Saccharomyces cerevisiae* (baker's yeast) is a classic and powerful model organism because it is easily cultivated and easily genetically modified. Yeast has been a key player in the understanding of many cellular processes, from human to plant biology [[1], [2], [3], [4]].

The functional analysis of proteins, for example, has been facilitated by the introduction of different yeast-based molecular assays, such as yeast complementation [5]. By using heterologous yeast complementation, many plant amino acid (AA) transporters have been identified [[6], [7], [8], [9], [10], [11]]. Notably, the complementation of the AA uptake-deficient yeast strain 22574d with the *Arabidopsis thaliana* AA transporter LYSINE HISTIDINE TRANSPORTER 1 (AtLHT1) helped to identify the specificity of the transporter with regard to the distinct AAs transported by AtLHT1 [8,[11], [12], [13]]. In addition to the functional characterization of individual proteins, the co-expression of two genes of interest in a yeast strain can provide insights into the regulation of the activity of a protein. For example, co-expression studies in yeast can reveal the impact on target protein activity when simultaneously expressed with a partner protein [3].

Traditionally, yeast complementation assays are performed on plates with solidified media, and although this method has been of paramount importance, difficulties with quantitative analysis are inherent. Hence, the results may be challenging to interpret, especially when differences in performance between targeted yeast strains are small. In other yeast-based methods like yeast two-hybrid (Y2H), the development of image-based assays opened the door to quantifying these solid media assays [14]. However, the interpretation and quantitative analysis of images of Y2H plates can be affected by, for instance, the spot size and shape, and may, therefore, be problematic.

Automation and high-throughput data generation are increasingly essential, and assays for yeast complementation using liquid culture growth combined with curve analysis have been published [15]. The benefit of this method is that it does not require any additional reagents other than standard solid media assays. Furthermore, the amount of hands-on work can be reduced by using, *e.g*., 96-well plates and automated incubation, shaking, and assessment processes. Spectrophotometric assessment of microbial growth, such as measurements of optical density at 600 nm (OD_600_), is commonly interpreted as absorbance at this wavelength [16]. However, for suspensions of microbial cells, most of the reduction in transmitted light is not due to absorbance, but rather to light scattering by particles whose sizes are comparable to, or larger than, the wavelength of the incident light. In such samples, particles scatter incident light in multiple directions, reducing the amount of light that reaches the detector and thereby causing an increase in the measured optical density, even in the absence of significant absorption [[16], [17], [18], [19]]. In addition, cell aggregation or clumping can further influence OD_600_ measurements, as aggregates scatter light differently than evenly dispensed cells and may introduce or increase noise in the spectrophotometric dataset.

In the current study, we present an alternative method for performing spectrophotometric liquid yeast assays and introduce a novel application of nephelometry in molecular biology. In contrast to spectrophotometry, which assesses culture density by measuring the reduction of intensity of transmitted light resulting from light scattering of cells [16,17], nephelometry directly measures the intensity of light scattered by particles in the sample, typically detecting scattered light at an angle (often around 90°) relative to the incident beam, which offers significant advantages in quantifying diverse microbial cultures [[20], [21], [22], [23]]. This sideways detection of the signal avoids interference from the transmitted light, because the detected signal arises from the light redistributed by scattering events rather than from light that continues in the forward direction.

Whereas traditional turbidity measurements are often expressed in Nephelometric Turbidity Units (NTU), nephelometric systems such as the one used here report cloudiness of a sample in Relative Nephelometric Units (RNU). RNU presents the relative intensity of detected scattered light, while turbidity measurements demonstrate the loss of intensity caused by scattering of the sample, similar to the absorbance measurement. Despite being relatively new in molecular biology, nephelometry has successfully been used to assess the growth of yeast, filamentous fungi, and algae, and has also been used to investigate the effect of antifungal drugs on yeast, as well as the effect of antibacterial compounds [20,21,[24], [25], [26], [27]]. Furthermore, nephelometry has been demonstrated to be a useful tool for quantifying polymeric nanoparticles [23]. Studies using algae and other aquatic eukaryotes compared the use of spectrophotometry and nephelometry. In the majority of experiments, nephelometry produced results comparable to, or better than, spectrophotometry in terms of reproducibility and sensitivity [21].

By comparing the ability of the two homologous plant AA transporters, AtLHT1 and PtrLHT1.2, to functionally complement an AA-deficient yeast mutant, we aimed to compare the performance and accuracy of different traditional yeast complementation methods with the use of nephelometry. In a previous study [10], no differences in mutant complementation ability between both plant transporters were shown, which is why this system was chosen for our comparative study. We compared traditional agar-based solid-phase complementation with liquid culture-based assays, evaluating the latter by either spectrophotometric scattering or RNU. To model and analyze the obtained growth curves assessed on the liquid assays, we used an in-house Python implementation of Growthcurver [28]. Fitted and smoothed growth curves were used to assess the lag time of growth of the culture, which is defined as the period following inoculation during which cells exhibit metabolic activity but undergo little to no cell division. In addition, the maximum slope time of the culture growth was determined, representing the point within the exponential phase at which the cells exhibit the highest growth rate.

Through a systematic evaluation of experimental approaches and a standardized, semi-automated solution to analyze data obtained from growth curves, we present a robust and reproducible way to assess and analyze yeast complementation in liquid assays using nephelometry.

## 2 Material and methods

### 2.1 Yeast strain and constructs

The *Arabidopsis thaliana* AA transporter AtLHT1 and the *Populus tremula L. x tremuloides Michx* (hybrid aspen) AA transporter PtrLHT1.2 were cloned into the yeast expression vector *pDRf1*, using the Gateway cloning system. Constructs and cloning strategies were constructed as described in Gratz et al. [10]. The yeast strain 22574d (*MATα ura3-1, gap1-1, put4-1, uga4-1)* [12], was transformed with *pDRf1:AtLHT1, pDRf1:PtrLHT1.2* or an empty *pDRf1:ccdB* vector as negative control. Positive transformants were selected on synthetic defined (SD) medium lacking uracil (SD-Ura) (Takara) and verified by colony PCR. All complementation assays were performed on yeast nitrogen base medium lacking AAs and ammonium sulfate (Thermo Fisher Scientific) and supplemented with either 3 mM l-citrulline (test condition; Thermo Fisher Scientific) or 10 mM ammonium sulfate (control condition; Thermo Fisher Scientific). The non-supplemented medium served as a negative control.

### 2.2 Solid media assay

All constructs were plated on nitrogen-free yeast base agar, lacking AAs and ammonium sulfate, supplemented with 3 mM l-citrulline or 10 mM ammonium sulfate or without additional nitrogen source (N-free). Pictures were taken after 5 days; 1 representative image is shown.

### 2.3 Liquid assay – spectrophotometer

A 3 ml pre-culture using SD-Ura was incubated overnight. The optical density (OD_600_) of each sample was measured (IMPLEN OD_600_). The cell volume for an OD_600_ = 0.1 in a final volume of 15 ml was calculated, transferred to a sterile tube, and centrifuged (13000 rpm, 15 s) to remove the old growth media. The supernatant was discarded, and the pellet was resuspended in the final growth media (N-free yeast base + 3 mM l-citrulline, N-free yeast base + 10 mM ammonium sulfate, or N-free yeast base) and used to inoculate a 15 ml main culture with a starting OD_600_ = 0.1. The OD_600_ of each culture was observed in cuvettes (Kartell Labware) twice per day for 7 consecutive days. The cultures were incubated at 30 °C and shaken at 200 rpm. Each OD_600_ measurement was performed in 3 technical replicates. The experiment was performed in 3 independent experimental runs. OD_600_ measurements were performed using culture-specific dilution factors to ensure the values fell within the linear range of the spectrophotometer. Following the OD_600_ measurements, the applied dilution factor was calculated from the measured value, and all reported OD_600_ values represent the corresponding undiluted culture density.

### 2.4 Liquid assay – nephelometer

A 3 ml pre-culture using SD-Ura was incubated overnight. The optical density (OD_600_) of each sample was measured (IMPLEN OD600). The cell volume for an OD_600_ = 0.1 in a final volume of 2 ml was calculated, transferred to a sterile tube, and centrifuged (13000 rpm, 15 s) to remove the old growth media. The pellet was resuspended in 2 ml sterile water. Followed by a 1:10 dilution step using the final growth media (N-free yeast base + 3 mM l-citrulline, N-free yeast base + 10 mM ammonium sulfate, or N-free yeast base) to reach a final OD_600_ = 0.01. 100 μl of the diluted yeast was pipetted into a sterile flat-bottom 96-well plate (Sarstedt) and covered with a transparent plastic foil (BioRad). The light scattering of each culture was measured every 10 min for 7 days, with a measurement interval of 20 s. The plates were incubated at 30 °C, and between measurements, the plate was orbitally shaken at 300 rpm with a shake width of 3 mm. The measurement, incubation, and shaking were performed fully automatically using Nephelostar (BMG LABTECH). The laser intensity was adjusted to 1 %, and the laser beam focus was set to 1.5 mm. The experiment was performed in 2 independent experimental runs, where each 96-well plate represented one experimental run and carried 3 biological replicates, including 3 technical replicates per construct and growth media. The sample position on the 96-well plate varied between experimental runs. In addition, non-inoculated growth media pipetted in triplicates served as a blank. The measurement was performed in Relative Nephelometric Units (RNU).

### 2.5 Data analysis

The Nephelometer raw data was extracted from the Omega MARS data analysis software version 4.01 R2 (BMG LABTECH) to an Excel file (Version 2503). The data obtained from both methods, nephelometry and spectrophotometry, underwent pre-processing before being analyzed. First, each measured time point was corrected for the blank value by subtracting the corresponding blank measurement for each growth condition from the recorded value. In a second step, the growth data of citrulline-grown constructs were normalized by subtracting the mean background growth of the *pDRf1:ccdB* negative control from the values of *pDRf1:AtLHT1* and *pDRf1:PtrLHT1.2*.

Before modelling, the growth curves were preprocessed as follows: 1) The minimum population size was found, and all values before the minimum in time were set to the minimum value. 2) The initial population size at time zero was set to 1 unit. 3) The growth curves were smoothed using a Savitzky–Golay filter [29] of order two with a window size covering 10% of the samples. 4) The growth curves of the technical replicates were averaged.

The growth data were modelled as natural population growth under limited resources, *i.e.*, using a logistic growth model. The in-house developed Python tool works much like the R package Growthcurver [28], and fits a logistic function, in the form

$$N\left(t\right)=\frac{K}{1+\left(\frac{K-N\left(0\right)}{N\left(0\right)}\right)e^{-rt}}\text{,}$$

using least squares, where N(t) is the population at time t, with N(0) thus the population at the start of the experiment, K is the carrying capacity (the maximum supported population), and r is the growth rate of the population. Initial values of K and N(0) were set to the maximum and minimum values of each growth data curve, respectively. The initial value of r was determined using the initial values of K and N(0) and solving for r in the logistic equation above, using the time of the maximum slope of the growth data, *i.e.*, as $r=\log\frac{K-N\left(0\right)}{N\left(0\right)}/\mu$, where μ is the time of the maximum slope. The values of K, N(0), and r were constrained to be positive.

From the logistic growth model, the time of the maximum slope of the logistic growth model was computed.

A Mann–Whitney *U* test (non-parametric rank test of equivalence; [30]) was used for the comparisons.

The Python code is available at [https://eur03.safelinks.protection.outlook.com/?url=https%3A%2F%2Fgithub.com%2Ftomlof%2Fgrowth-curve-analysis&data=05%7C02%7Cregina.gratz%40slu.se%7Cd7d03c7697c9474fd5ef08de9027dfaf%7Ca3b5f0710e4947a0a40e9b7c9c4d647e%7C1%7C0%7C639106698017473726%7CUnknown%7CTWFpbGZsb3d8eyJFbXB0eU1hcGkiOnRydWUsIlYiOiIwLjAuMDAwMCIsIlAiOiJXaW4zMiIsIkFOIjoiTWFpbCIsIldUIjoyfQ%3D%3D%7C0%7C%7C%7C&sdata=MDvNE7rUS4l56mGnQlghAYf1Cjrlw6fGv%2F9n%2BdnpoyY%3D&reserved=0].

## 3 Results

### 3.1 Solid yeast complementation assay

**Fig. 1:**
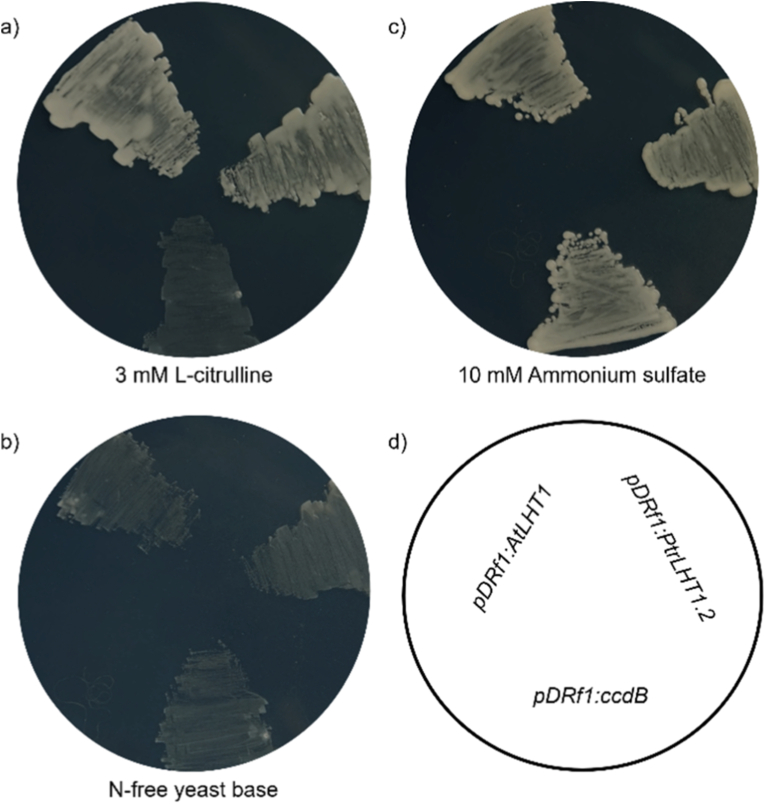
*Saccharomyces cerevisiae* mutant complementation on solid media. The AA uptake-deficient yeast strain 22574D was transformed with vectors carrying AA transporter *AtLHT1*, *PtrLHT1.2,* or the empty *pDRf1:ccdB* vector as a negative control. The constructs were grown on N-free yeast base supplemented with 3 mM l-citrulline (a), N-free yeast base (b), or N-free yeast base supplemented with 10 mM ammonium sulfate (c). Visualization of the plating scheme (d). Plates were incubated at 30 °C, and pictures were taken after 5 days. The experiment was repeated 3 times; 1 representative image is shown.

Yeast strain 22574d [12], deficient in the uptake of l-citrulline, proline, and γ-aminobutyric acid (GABA), was transformed with the Arabidopsis AA transporter AtLHT1, poplar PtrLHT1.2, or the empty *pDRf1:ccdB* vector as negative control (Fig. 1). When plating single colonies on N-free yeast media supplemented with l-citrulline, only yeast expressing either *AtLHT1* or *PtrLHT1.2* showed growth. Both transporters displayed comparable growth at the end of the incubation time, however, quantification of growth was not possible. Yeast transformed with the empty vector did not show any growth (negative control), the empty vector alone was hence not able to complement the yeast AA uptake phenotype (Fig. 1a). Yeast colonies plated either on N-free medium (Fig. 1b) as negative control, or on media containing ammonium sulfate (Fig. 1c) as growth control, showed no growth and growth respectively.

### 3.2 Liquid yeast complementation assays

**Fig. 2:**
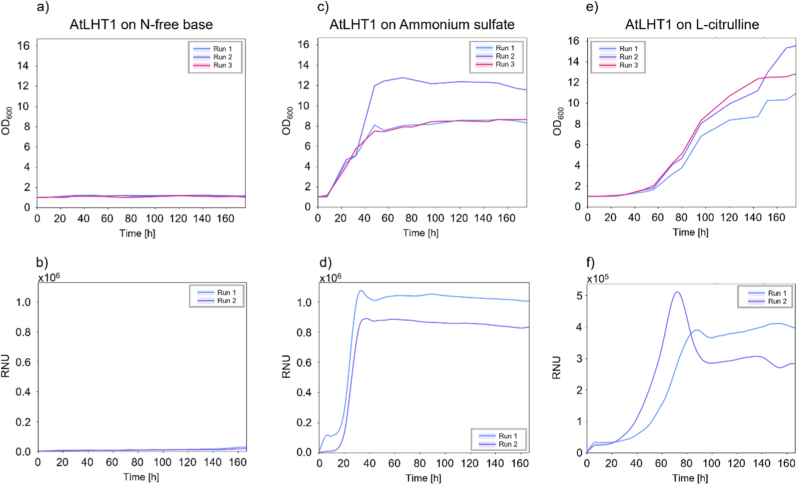
*Saccharomyces cerevisiae* mutant complementation with the AA transporter *LHT1* from *Arabidopsis thaliana* (*AtLHT1*) in liquid media using spectrophotometry (a, c, e) or nephelometry (b, d, f). Yeast cells were grown either in N-free media (a, b), N-free media supplemented with 10 mM ammonium sulfate (c, d), or N-free media supplemented with 3 mM l-citrulline (e, f) for 180 h. Three replicates were used for spectrophotometry, while two replicate runs, each comprising three technical replicates, were used for nephelometry.

In order to quantify specific growth characteristics, liquid-, instead of plate-based, yeast complementation assays can be employed. The read-out of the respective growth curve is typically assessed via spectrophotometry, measuring optical density at wavelength 600 nm (OD_600_). To establish our assays and to control for reproducibility of individual repetitions, single colonies of transformed yeast expressing *AtLHT1* were cultivated in either N-free yeast base medium, medium containing ammonium sulfate, or l-citrulline. The negative control (yeast grown in N-free yeast media; Fig. 2a) and the positive control (yeast grown on ammonium sulfate; Fig. 2c) showed no growth and growth, respectively. Notably, run two displayed a slightly different growth pattern compared to the other two runs, with an extended exponential phase before reaching the stationary phase (Fig. 2c). Yeast that expressed *AtLHT1* showed growth when l-citrulline was present in the growth media (Fig. 2e). The results of all three individual runs are shown in Fig. 2. Using the same setup, nephelometric measurements produced similar results with no growth on the N-free media and significant growth on ammonium sulfate media (Fig. 2b and d, respectively). However, growth curves of yeast expressing *AtLHT1* on media containing l-citrulline displayed a peak in scattering intensity (RNU) followed by a decline and a plateau (Fig. 2f).

**Fig. 3:**
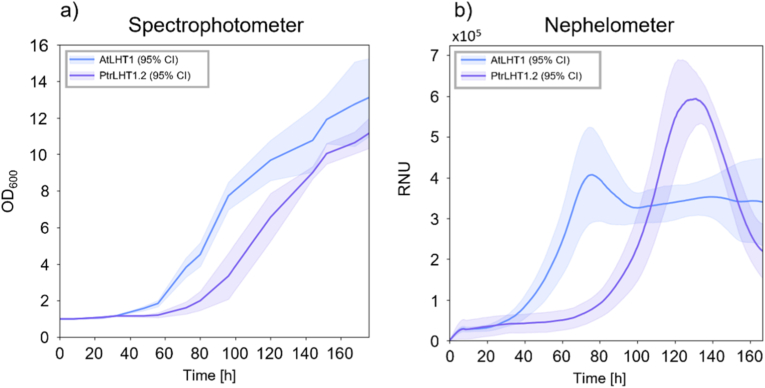
*Saccharomyces cerevisiae* mutant complementation with the AA transporter *LHT1* from *Arabidopsis thaliana* (*AtLHT1*) or the AA transporter *LHT1.2* from Poplar (*PtrLHT1.2*) on liquid media using spectrophotometry (a) or nephelometry (b). Yeast cells were grown in N-free media supplemented with 3 mM l-citrulline for 180 h. Curves represent the average of three and two replicates (each comprising 3 technical replicates) for spectrophotometry and nephelometry, respectively. Shaded areas represent the 95% confidence interval (CI).

In a next step, we compared the growth of yeast complemented with either *AtLHT1* or *PtrLHT1.2* and cultivated on l-citrulline media. Both were previously shown to complement the yeast mutant to the same extent [10]. Spectrophotometry (Fig. 3a) and nephelometry (Fig. 3b) displayed strong yeast growth. Interestingly, differences in the respective growth curve characteristics could be detected. Growth curves recorded with the spectrophotometer show a clear lag phase followed by an exponential phase, before gradually approaching a plateau. Yeast complemented with *AtLHT1* enters the exponential phase earlier. Growth curves recorded by the nephelometer begin with a short lag phase and then enter an exponential phase. Instead of reaching a stable plateau phase, they reach a pronounced maximum and subsequently decline. Also here, yeast complemented with *AtLHT1* enters the exponential phase and respective peak earlier.

To better compare growth characteristics between yeast complemented with *AtLHT1* and *PtrLHT1*.*2* in both setups, we next calculated the maximal slope time.

**Fig. 4:**
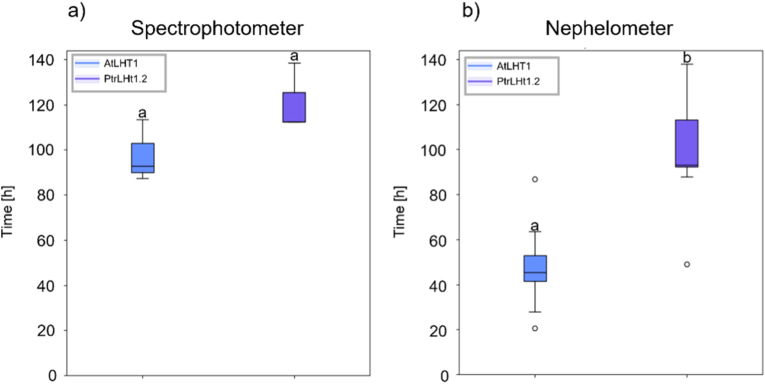
Time of maximum growth rate (maximum slope time) for yeast complementation with either *AtLHT1* (blue boxes) or *PtrLHT1.2* (purple boxes) as assessed through spectrophotometry (a) or through nephelometry (b). Different letters close to whiskers represent statistical difference between constructs using Mann-Whitney *U* test (p ≤ 0,001).

No difference in performance of the two constructs could be detected with spectrophotometry (Fig. 4a), while a clear statistically significant difference in time of maximum growth was detected between the constructs with nephelometry (Fig. 4b; p ≤ 0.001).

## 4 Discussion

In the current study, we investigated the feasibility of using nephelometry for the analysis of heterologous yeast complementation. The classic agar-based method (Fig. 1) provides visual information on the growth of yeast strains and has been of paramount value, not the least in yeast complementation studies. However, subtle differences in performance are difficult to detect with this method. Liquid-based methods (Fig. 2) are therefore better suited for quantifying growth, and subtle differences in the performance may hence become detectable. Furthermore, there is less hands-on work in liquid culture experiments, no additional chemicals or reagents are needed, and growth media volumes can also be lower.

The growth of yeast in liquid culture media is usually assessed using OD_600_. Cell growth in the media leads to a decline in transmitted light, not as in the case of chemical solutions through absorbance in the liquid media, but through light scattering. The fundamental difference between spectrophotometry and nephelometry is that the former measures a decrease in transmission due to light scattering, while the latter measures the increase in scattering as yeast cells proliferate. This difference is of particular importance when assessing the growth of, *e.g*., filamentous fungi or other organisms forming aggregates, and hence, for studies on such organisms, the advantage of using nephelometry is significant [20,21,24,25].

### 4.1 Nephelometer use is suited for yeast complementation assays

In the current study, spectrophotometer as well as nephelometer data mirrored the results of the solid media experiment (Fig. 1, Fig. 2), showing that the deficient yeast phenotype grew on l-citrulline when complemented with the AA transporter LHT1 (AtLHT1) (Fig. 2e and f). Control experiments using N-free media displayed no increase in light scattering using either spectrophotometry or nephelometry (Fig. 2a and b). Similarly, the positive control with complemented yeast strains all grew at similar rates on ammonium sulfate (Fig. 2c and d). These results support the notion that spectrophotometry and nephelometry can be utilized to analyze yeast growth in yeast complementation assays.

At the same time, we noticed that the growth curves differ in shape depending on the method. While spectrophotometer-generated curves show a more classical lag and exponential phase, slowly transitioning into a stationary phase (Fig. 2c–e), we could observe a peak in nephelometry-generated growth curves, which then declined before transitioning into a plateau (Fig. 2d–f).

### 4.2 Nephelometer use might be more sensitive, allowing to highlight small differences in growth kinetics of yeast expressing *LHT1*

We further compared the growth of the two yeast mutants, complemented either with *AtLHT1* or *PtrLHT1.2*, using either spectrophotometry or nephelometry (Fig. 3a and b, respectively). The two LHT1 homologues share a high identity in the nucleotide and AA sequence and have been shown to exhibit similar characteristics in terms of activity and specificity [10]. The close homology suggests the two constructs should perform equally well in the yeast complementation system, which was previously shown on a solid-plate assay [10]. Spectrophotometric assessment did not reveal any significant differences between the ability of the two homologues to rescue the yeast mutant phenotype (Figs. 3a–4a). However, nephelometric measurements pointed to a significant difference in the maximum slope time of growth of cultures (Figs. 3b–4b). Maximum slope time is related to the length of the lag phase, which is a well-known metric for assessing the performance of microbes in different media [31]. Alterations in the lag phase duration can reflect differences in the ability of cells to adapt to nutrient availability and changing environmental conditions [32,33]. A shorter lag phase is generally associated with a faster adaptation, a competitive advantage, and an earlier onset of exponential growth. We can conclude that both homologues can restore growth of the mutant yeast, indicating that both genes can functionally complement the missing AA uptake ability, as seen in Gratz et al. (2021) [10]. Yeast expressing the Arabidopsis *LHT1* gene displayed a shorter lag phase, as shown by a significantly reduced maximum slope time compared with yeast expressing the Poplar *LHT1.2* gene (Fig. 3). It might be that AtLHT1 transports l-citrulline more effectively in yeast, allowing cells to take up nutrients faster. It is also possible that AtLHT1 might be expressed at higher levels in yeast or that the protein is more stable compared to PtrLHT1.2. It would also be interesting to trace the incorporation of the heterologous proteins into the plasma membrane to see if, potentially, PtrLHT1.2 is, in part, mislocated.

### 4.3 Methodological considerations

We are comparing a fully automated nephelometric setup with a manually executed spectrophotometric assay. Hence, comparing the two methods introduces certain potential limitations, since the liquid-based methods differ substantially in their experimental set-up: the chosen difference in starting culture density could be expected to affect the experimental outcome. Cultures measured with the spectrophotometer started with a higher initial OD_600_ (0.1) than those monitored with the nephelometer (0.01). In principle, the higher starting density should result in a shorter lag phase. However, the observed results presented in Fig. 3, Fig. 4 show the opposite trend. To exclude effects introduced by, *e.g.*, culture conditions or the mutant strain, in a future study, different yeast strains or adjusted starting densities should be evaluated.

Besides, nephelometric measurements are performed in a closed, automated system that integrates incubation, shaking, and data acquisition, enabling continuous, minimal invasive monitoring of cultures. In the present study, cultures were shaken almost continuously, with only brief interruptions during measurements conducted every 10 min for 20 s. This represents a shorter interruption of shaking than reported in earlier nephelometric studies, which typically performed a 5-min shaking cycle every 10 min [20,21]. Importantly, during these brief measurement intervals, incubation conditions such as temperature were maintained, minimizing environmental fluctuations. In contrast, spectrophotometric measurements required manual sampling of cultures grown in flasks, shaken in incubators. The handling of the samples might introduce stress factors, including temperature changes, alterations in cell volume, and gradual shifts in nutrient availability. The results presented in Fig. 2, Fig. 3 demonstrate that these effects are unlikely to alter the overall qualitative trends, however, they might contribute to more subtle differences in growth kinetics between measurement techniques. In particular, the uninterrupted and minimal invasive monitoring by the nephelometer might have enhanced sensitivity to subtle growth dynamics, allowing a clearer discrimination between the two constructs, AtLHT1 and PtrLHT1.2, when it comes to the maximum slope time.

Besides potential effects that sample handling might have, different shapes in the generated curves might be due to differences in the measurement principles of both methods. As mentioned before, spectrophotometry measures light that can pass through a culture. With increasing cell density, more light is absorbed and scattered, and the loss of transmitted light will increase. This leads to a typical growth curve defined by a lag, exponential, and stationary phase. The nephelometer measures the scattered light at a specific angle. While cell density is still moderate during the lag and exponential phase, the light scatters independently. However, when the culture becomes very dense, multiple scattering might occur, where photons are scattered by more than one cell, and hence the signal becomes amplified. This might lead to the observed peak at the end of the exponential phase (Fig. 2d–f, and Fig. 3b). When the culture reaches the stationary phase, the cell number stabilizes and so does the signal. Hence, after a signal decline, a plateau similar to the spectrophotometer measurements is established. The differences in growth curve dynamics between the instruments might hence be influenced by the optical physics underlying how the signal is generated, rather than biological differences between the samples. Given those differences, we are presenting a study with certain limitations that need to be addressed in a follow-up study.

Nevertheless, we could identify relative differences between the performance of AtLHT1 and PtrLHT1.2 when it comes to their ability to complement an AA-uptake-deficient yeast mutant strain. None of the above-mentioned influences regarding sample handling or differences between the methods would explain this difference. Hence, we speculate that AtLHT1 is more efficient in the uptake of l-citrulline in the yeast system, which points to the fact that both homologues differ in their efficacy. This might be due to the slight differences in AA identity when comparing both sequences. In future experiments, it will be important to investigate if those diverging AAs might carry functional importance. In an unpublished study, we point-mutagenized individual AAs in the AtLHT1 sequence and interestingly found similar differences in maximum slope time when comparing those mutagenized versions of AtLHT1 (Tünnermann et al., *in prep*).

## 5 Conclusions

In this study, we evaluated the potential of nephelometry for analyzing growth of yeast cells in mutant complementation experiments. A comparative study using the ability of the two AA transporters, AtLHT1 and PtrLHT1.2, to complement an AA-uptake-deficient yeast strain was performed to compare nephelometry to traditional approaches, such as spectrophotometric approaches. We can conclude that i) nephelometry is able to record cell growth in a yeast complementation study, ii) nephelometry is more sensitive to small changes in cell growth characteristics, which, however, might be due to particle properties, iii) the *LHT1* homologues differ in functional efficacy to complement yeast mutants, deficient in taking up AA.

These findings provide a promising basis for future studies, which will help to further validate and establish nephelometry for yeast analysis. In addition, further biological assays will be needed to investigate the differences between both homologues to conclude whether potential differences in sequence identity can explain our observations and whether individual AAs carry a functional importance.

## Funding

This work was supported by the 10.13039/501100007067Kempe Foundations (JCK-2122 to RG) and by the 10.13039/501100004063Knut and Alice Wallenberg Foundation (2018.0259 to TN).

## CRediT authorship contribution statement

**Laura Tünnermann:** Data curation, Formal analysis, Investigation, Methodology, Project administration, Validation, Writing – original draft, Writing – review & editing. **Justine Colou:** Conceptualization, Data curation, Formal analysis, Investigation, Methodology, Project administration, Writing – review & editing. **Torgny Näsholm:** Conceptualization, Formal analysis, Funding acquisition, Investigation, Methodology, Writing – original draft, Writing – review & editing. **Tommy Löfstedt:** Conceptualization, Data curation, Formal analysis, Writing – review & editing. **Regina Gratz:** Conceptualization, Data curation, Formal analysis, Funding acquisition, Investigation, Methodology, Project administration, Resources, Supervision, Writing – original draft, Writing – review & editing.

## Declaration of competing interest

The authors declare that they have no known competing financial interests or personal relationships that could have appeared to influence the work reported in this paper.

## Data Availability

Data will be made available on request.
